# Pulmonary Embolism and Myocardial Infarction With Non-obstructive Coronary Arteries in Immune Thrombocytopenia: Unmasking Underlying Antiphospholipid Syndrome

**DOI:** 10.7759/cureus.100731

**Published:** 2026-01-04

**Authors:** Muhammad Shahzeb, Faiqa Jabeen Naeem, Kiran Naz, Muhammad Irfan, Nawaid Ahmad, Nawal Rafiq, Ijaz Ul Haq

**Affiliations:** 1 Endocrinology and Diabetes, Shrewsbury and Telford Hospital NHS trust, Telford, GBR; 2 Acute Medicine, Queen Elizabeth Hospital Birmingham, Birmingham, GBR; 3 Acute Medicine, Shrewsbury and Telford Hospital NHS Trust, Shrewsbury, GBR; 4 Internal Medicine, Princess Royal Hospital, Telford, GBR; 5 Respiratory and Acute Medicine, The Shrewsbury and Telford Hospital NHS Trust, Telford, GBR; 6 Accident and Emergency, Rehman Medical Institute, Peshawar, PAK; 7 Medicine, Northwest General Hospital and Research Center, Peshawar, PAK

**Keywords:** antiphospholipid syndrome (aps), autoimmune thrombocytopenia, hypercoagulable state, immune thrombocytopenia (itp), myocardial infarction with non-obstructive coronary arteries (minoca), paradoxical thrombosis, patent foramen ovale (pfo), pulmonary embolism, thromboembolism

## Abstract

This case report presents the clinical scenario of a 35-year-old male patient who experienced chest pain due to a combination of pulmonary embolism (PE) and myocardial infarction with non-obstructive coronary arteries (MINOCA), concurrently while undergoing treatment with avatrombopag for immune thrombocytopenia (ITP). His investigations included a CT pulmonary angiogram that confirmed a PE, a coronary angiography which was normal, a cardiac MRI which showed evidence of subendocardial infarct, and a CT coronary angiogram, which was normal. His unique presentation with these findings prompted further investigations, which revealed an undiagnosed antiphospholipid syndrome (APS) alongside a patent foramen ovale (PFO). Hence, the paradoxical thrombotic incidents were precipitated by this unique diagnosis. After establishing the diagnosis, our patient was commenced on warfarin, and his treatment protocol for ITP was changed to a different drug. He remains under haematology follow-up.

## Introduction

Immune thrombocytopenia (ITP) is an autoimmune condition characterized by the production of antibodies that target platelets, resulting in their early destruction and a lower platelet count. While bleeding episodes are frequently observed in individuals with ITP, there are rare instances where a thrombotic event may arise. Such occurrences typically suggest the presence of an underlying prothrombotic disorder rather than being a direct consequence of ITP or its treatment [1,2]. 

Myocardial infarction with non-obstructive coronary arteries (MINOCA) describes a scenario where patients exhibit clinical signs and biochemical markers indicative of myocardial injury but do not have significant blockages in their coronary arteries. The mechanisms behind MINOCA are diverse and can encompass issues like coronary embolism, vessel spasm, inflammation, or dysfunction of the microvasculature [3]. Understanding the root causes is crucial as treatment strategies and long-term management depend on identifying the underlying pathology.

This case report details the situation of a 35-year-old male patient who was being treated with avatrombopag for immune thrombocytopenia (ITP). During his treatment, he sustained both a pulmonary embolism and MINOCA simultaneously. Further investigations revealed an undiagnosed antiphospholipid syndrome (APS) alongside a patent foramen ovale (PFO), which played a role in the unexpected thrombotic incidents. Although both venous and arterial thromboembolism may arise as a direct result of ITP or its treatment, the presence of APS in our patient showcases the need to consider a wider differential diagnosis in similar situations, as it will necessitate a change in management plan.

## Case presentation

We hereby describe the case of a 35-year-old man with idiopathic thrombocytopenic purpura (ITP). He was initially diagnosed with ITP in January 2017 in Romania, and was treated with a course of high-dose corticosteroids orally as well as a single dose of intravenous immunoglobulin (IVIG). After achieving complete remission, he has been closely monitored by a haematologist in the UK.

In January 2025, the patient experienced a relapse of ITP, where he presented with a petechial rash and a platelet count of 30 ×10⁹/L. He was prescribed prednisolone at a dosage of 60 mg daily, to which he initially responded well. However, during his follow-up appointment at the haematology clinic in April 2025, it became evident that his response to the steroid treatment was only temporary, leading to steroid failure. Given the significant thrombocytopenia and unsuccessful steroid response, treatment with avatrombopag was initiated for prolonged platelet support.

First admission - July 2025

In July 2025, the patient presented to the Emergency Department with complaints of sudden onset chest pain, pleuritic, radiating to the back, associated with shortness of breath on exertion and mild orthopnoea. He did not complain of any cough or haemoptysis. On arrival, his ECG demonstrated ST-segment depression in leads V4-V6, III, and aVF. Laboratory investigations revealed a troponin T level of 337 ng/L, D-dimer of 912 ng/mL, and a platelet count of 205 ×10⁹/L.

His acute presentation raised suspicion of a myocardial ischaemia, and therefore, he was referred to the tertiary cardiology unit nearby for an invasive coronary angiogram.

**Figure 1:**
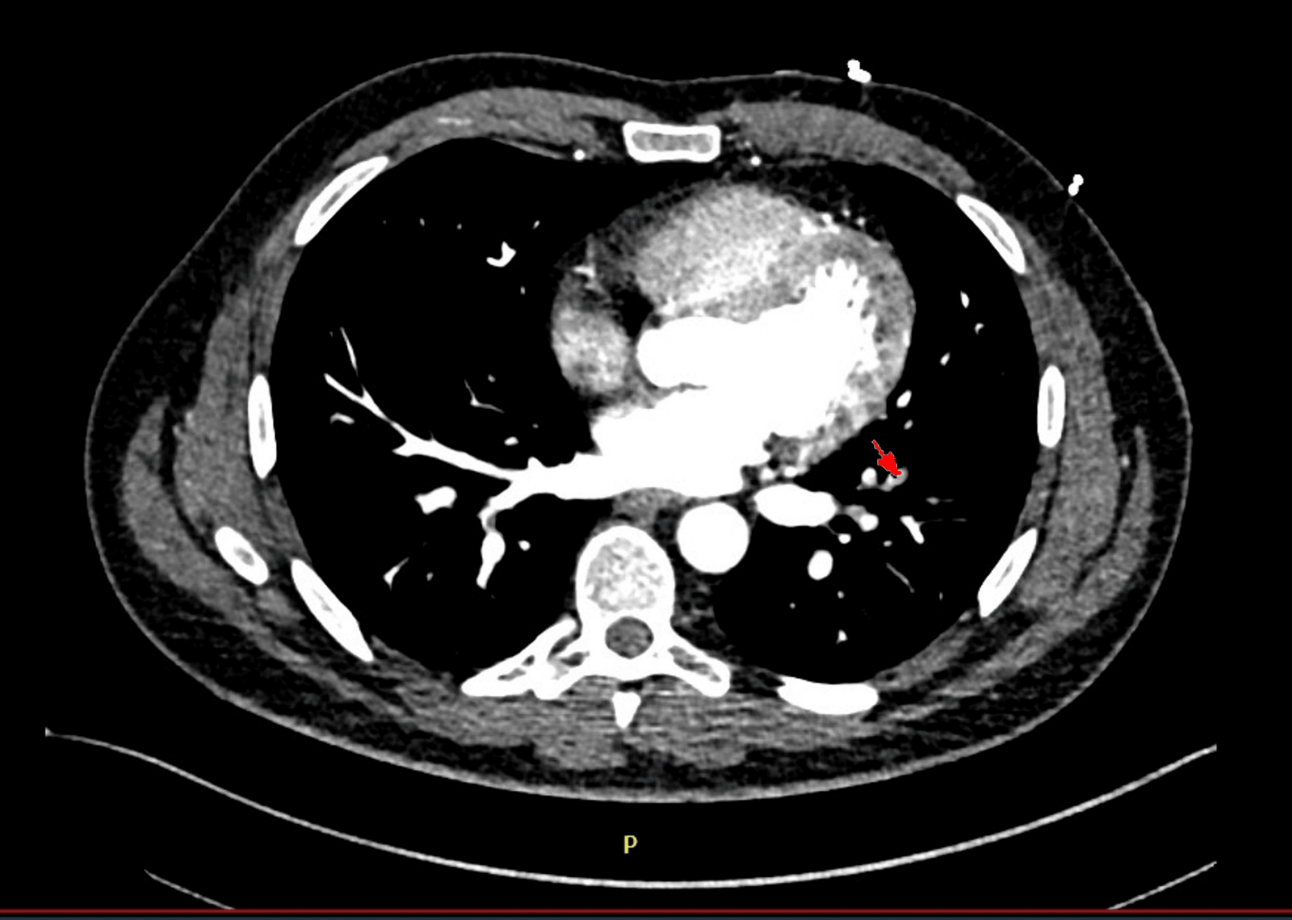
CT pulmonary angiogram Arrow pointing towards a low-volume pulmonary embolism in the distal segment of the left lower lobe.

He had a CT pulmonary angiogram (CTPA) (Figure 1), which confirmed the presence of a low-volume pulmonary embolism in the distal segment of the left lower lobe.

He had a coronary angiography at the tertiary hospital, which showed non-obstructing coronary arteries. A cardiac MRI was performed and demonstrated small subendocardial infarctions consistent with myocardial infarction with non-obstructive coronary arteries (MINOCA). 

In view of the above findings, he was started on a loading dose of apixaban for seven days (10 mg twice daily) followed by a lifelong course at 5 mg twice daily. Meanwhile, his case was discussed with his responsible haematologist to ascertain whether these thrombotic events could be related to his underlying diagnosis of ITP or his treatment. It was under the impression that this thrombotic event was unlikely to be related to ITP or avatrombopag treatment, given the normal platelet count at the time of his event. They recommended further investigations, particularly looking to exclude antiphospholipid syndrome; thereby, an anti-phospholipid screening was done.

He was referred back to us, needing close monitoring by the haematology team. Over the following weeks, his platelet count fluctuated, dropping to as low as 2x10^9^/L in September 2025, though he remained asymptomatic. Haematology managed this by balancing avatrombopag dosing, alternating between holding and restarting treatment to maintain stable platelet levels.

In view of the thrombotic event involving both venous and arterial systems, it was felt prudent to consider doing a bubble echocardiogram as an outpatient. A bubble echocardiogram was performed by the tertiary cardiology centre, which confirmed the presence of a patent foramen ovale.

Second admission - September 2025

In September 2025, the patient re-presented with chest pain radiating to the back, associated with dyspnoea on exertion. His initial ECG only showed a normal sinus rhythm. Laboratory investigations revealed a troponin T level of 25 ng/L and a platelet count of 242 ×10⁹/L. 

**Figure 2:**
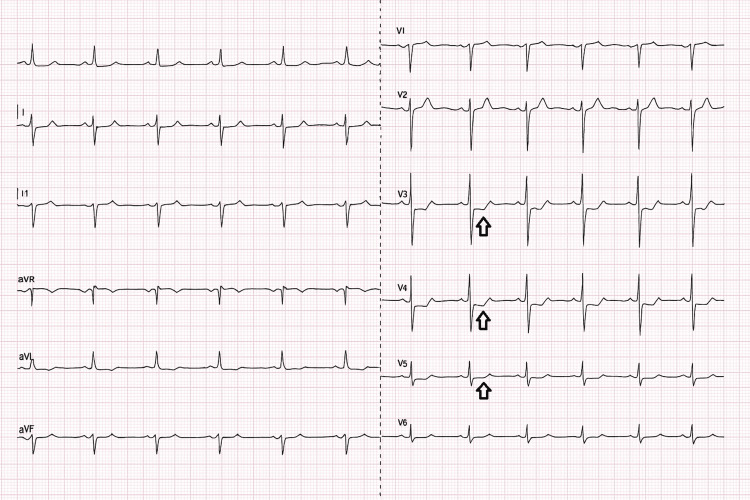
Repeat ECG on second admission. Repeat ECG showing ST depression in V3-V5.

A CTPA was repeated, which did not demonstrate a new pulmonary embolism. He was observed for 72 hours, during which he continued to experience recurrent chest pains. A repeat ECG (Figure 2) at this point showed ST-segment depression in leads V3-V5, and there was an incremental rise in his troponin T level to 331 ng/L, prompting a re-discussion with the cardiology team at the tertiary hospital. 

**Figure 3:**
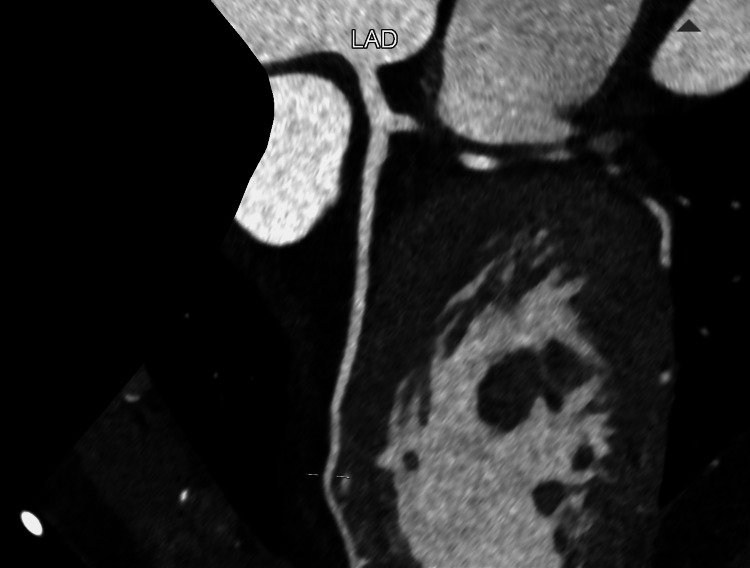
CT coronary angiogram (CTCA) showing left anterior descending coronary artery. CTCA showing the lumen is well-opacified with contrast with no visible narrowing or calcified plaques.

**Figure 4:**
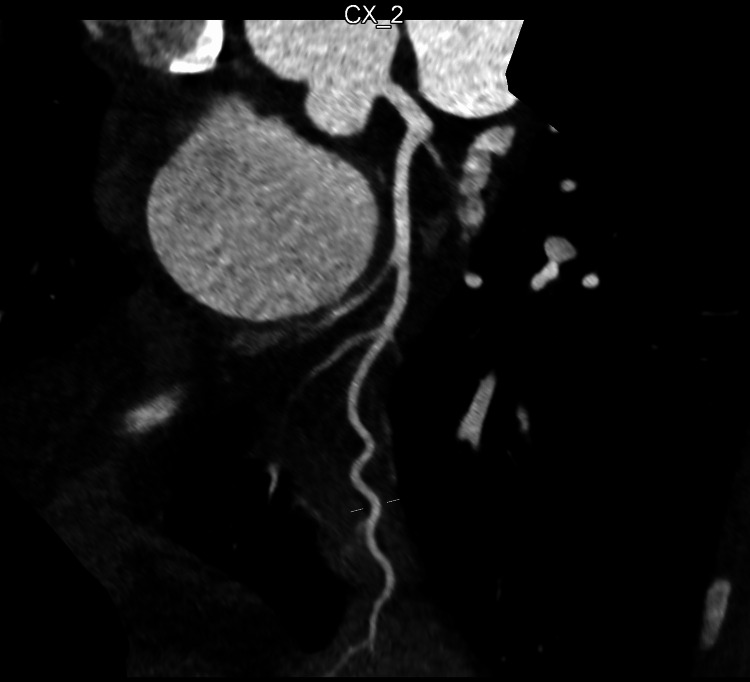
CT coronary angiogram showing left circumflex coronary artery. Normal left circumflex artery with no evidence of coronary atherosclerosis.

He was once again transferred to the tertiary cardiology centre for further investigation. A CT coronary angiogram (CTCA) (Figures 3, 4) revealed a coronary calcium score of 0 and no evidence of coronary artery disease. 

Given the persistent myocardial injury without obstructive coronary lesions, a diagnosis of MINOCA was confirmed. Subsequent autoimmune testing identified positive APS antibodies, confirming a diagnosis of antiphospholipid syndrome (APS). In the context of pre-existing ITP, this was interpreted as APS-associated MINOCA.

**Table 1:** Summary of Laboratory Investigations Autoimmune testing revealed strongly positive anticardiolipin IgG antibodies on two separate occasions, 12 weeks apart, fulfilling the laboratory criteria for antiphospholipid syndrome (APS). Additionally, B2-glycoprotein IgG was also elevated, providing further serological evidence for APS. Lupus anticoagulant could not be performed due to therapeutic warfarin. Together, these findings supported a diagnosis of APS-associated myocardial infarction with non-obstructive coronary arteries (MINOCA) in the context of pre-existing immune thrombocytopenia (ITP).

Test	Result	Reference Range	Clinical Interpretation
Platelet count - (ITP relapse)	30x10^9 ^/L	150-400 x10^9^/L	Severe thrombocytopenia
Platelet count (first admission)	205x10^9 ^/L	150-400 x10^9^/L	Normal
Platelet count - Post-discharge	24x10^9^/L	150-400 x10^9^/L	Drop after holding Thrombopoietin Receptor Agonist
Platelet count (Second admission)	242x10^9^/L	150-400 x10^9^/L	Normal
D-dimer - First admission	912 ng/mL	<500 ng/mL	Elevated; prompted pulmonary embolism workup
Troponin T - First admission	337 ng/L	<14 ng/L	Elevated
Troponin T - Second admission (serial measurements)	25 ->62 ->86 -> 94 ->331 ng/L	<14 ng/L	Rising pattern consistent with MINOCA
Anticardiolipin IgG antibody (first set)	249 GPL-U/mL	<10 GPL-U/mL	Strongly positive; APS criterion
Anticardiolipin IgG antibody (repeat)	300 GPL-U/mL	<10 GPL-U/mL	Persistent high-titer positivity at 12 weeks
Beta-2 Glycoprotein I IgG antibody	61.0 U/mL	<7 U/mL	Strongly positive; APS criterion
Lupus anticoagulant	Not performed		Could not be tested due to warfarin therapy
International Normalized Ratio (INR)	2.2 (target 2-3)		Within range

Management and follow-up

Following multidisciplinary discussion between both cardiology and haematology teams, his anticoagulation was switched from apixaban to warfarin, with a target international normalized ratio (INR) level designated between 2 and 3.

He continues under regular haematology supervision and switched to eltrombopag 50 mg once daily (Monday to Thursday), with dosing adjusted according to platelet counts. The medication is withheld when platelet counts exceed 250 ×10⁹/L to avoid thrombocytosis. He remains clinically stable on this regimen, with no recurrence of chest pain, thrombotic, or bleeding complications.

## Discussion

This case describes a paradoxical thrombotic incident where a patient with ITP on thrombopoietin receptor agonist treatment suffered from a thrombotic sequelae of pulmonary and coronary circulation. Thrombosis in ITP can result from several mechanisms, including immune-mediated endothelial activation, microparticles of platelets, and thrombopoietin agonist-related effects [4]. However, platelet values were normal during both episodes, and the positive antibody profile for anti-phospholipid antibodies indicates that the predominant pathology causing this event was related to APS.

Antiphospholipid syndrome is an autoimmune prothrombotic condition characterised by recurrent venous and arterial thromboses in the presence of anti-phospholipid antibodies [5]. APS is also a recognised cause of MINOCA and coronary embolism, particularly in young adults without atherosclerotic risk factors [6]. The detection of PFO also lends support to a possible paradoxical embolism mechanism - thrombi passage from venous to arterial circulation via an intracardiac shunt [7]. This explains the presence of both pulmonary embolism and coronary infarction at the same time.

Thrombosis management in ITP is tricky due to the inherent risk of bleeding. Anticoagulation is still considered fundamental for APS, having historically favoured warfarin over direct-acting oral anticoagulants (DOACs) due to a reported inferior outcome with DOACs in APS [8].

Our case highlights the importance of implementing APS screening in ITP patients with thromboembolic events and echocardiography to screen for intracardiac shunts in patients with paradoxical embolism. The rapid recognition of an underlying cause has an important impact on the long-term management of MINOCA, and clinicians must remain vigilant for these patients who present with recurrent episodes of chest pain despite being treated for thrombotic incidents,as finding an alternative diagnosis helps change the pathway to management and achievement of better long-term outcomes.

## Conclusions

Patients with immune thrombocytopenia (ITP) might unexpectedly have thrombotic complications due to prior or co-occurring prothrombotic conditions. This case highlights the significance of assessing for antiphospholipid syndrome and cardiac shunts in ITP subjects with thromboembolic events or MINOCA (myocardial infarction with non-obstructive coronary arteries). An interdisciplinary approach involving specialists from hematology, cardiology, and rheumatology is essential to advance both diagnosis and management.
